# Herbicide and Cytogenotoxic Activity of Inclusion Complexes of *Psidium gaudichaudianum* Leaf Essential Oil and *β*-Caryophyllene on 2-Hydroxypropyl-*β*-cyclodextrin

**DOI:** 10.3390/molecules28155909

**Published:** 2023-08-06

**Authors:** Luiza Alves Mendes, Loren Cristina Vasconcelos, Milene Miranda Praça Fontes, Geisiele Silva Martins, Aline dos Santos Bergamin, Matheus Alves Silva, Rafael Resende Assis Silva, Taíla Veloso de Oliveira, Victor Gomes Lauriano Souza, Marcia Flores da Silva Ferreira, Róbson Ricardo Teixeira, Renata Pereira Lopes

**Affiliations:** 1 Departament of Chemistry, Federal University of Viçosa (UFV), Av. Peter Henry Rolfs, s/n, Campus Universitário, Viçosa 36570-000, MG, Brazil; robsonr.teixeira@ufv.br; 2 Department of Biology, Federal University of Espírito Santo (UFES), Alto Universitário, s/n, Guararema, Alegre 29500-000, ES, Brazil; loren-vasconcelos@hotmail.com (L.C.V.); milenemiranda@yahoo.com.br (M.M.P.F.); martinsgeisiele@gmail.com (G.S.M.); 3 Department of Agronomy, Federal University of Espírito Santo (UFES), Alto Universitário, s/n, Guararema, Alegre 29500-000, ES, Brazil; alinebergamin258@hotmail.com (A.d.S.B.); alvesmatheuss21@gmail.com (M.A.S.); mfloressf@gmail.com (M.F.d.S.F.); 4 Departament of Food Materials Science and Engineering, Federal University of São Carlos (UFSCar), Rod. Washington Luiz, s/n, São Carlos 13565-905, SP, Brazil; rafael.resendeas@gmail.com; 5 Department of Food Technology, Federal University of Viçosa, Viçosa 36570-900, MG, Brazil; taila.oliveira@ufv.br; 6 International Iberian Nanotechnology Laboratory (INL), Av. Mestre José Veiga s/n, 4715-330 Braga, Portugal; 7 MEtRICs, CubicB, Departament of Chemistry, NOVA School of Science and Technology (FCT NOVA), University Nova de Lisboa, Campus de Caparica, 2829-516 Caparica, Portugal

**Keywords:** natural product, araçá, inclusion complex, biological activity, bioherbicide

## Abstract

The present investigation aimed to develop inclusion complexes (ICs) from *Psidium gaudichaudianum* (GAU) essential oil (EO) and its major compound *β*-caryophyllene (*β*-CAR), and to evaluate their herbicidal (against *Lolium multiflorum* and *Bidens pilosa*) and cytogenotoxic (on *Lactuca sativa*) activities. The ICs were obtained using 2-hydroxypropyl-*β*-cyclodextrin (HP*β*CD) and they were prepared to avoid or reduce the volatility and degradation of GAU EO and *β*-CAR. The ICs obtained showed a complexation efficiency of 91.5 and 83.9% for GAU EO and *β*-CAR, respectively. The IC of GAU EO at a concentration of 3000 µg mL^−1^ displayed a significant effect against weed species *B. pilosa* and *L. multiflorum*. However, the *β*-CAR IC at a concentration of 3000 µg mL^−1^ was effective only on *L. multiflorum*. In addition, the cytogenotoxic activity evaluation revealed that there was a reduction in the mitotic index and an increase in chromosomal abnormalities. The produced ICs were able to protect the EO and *β*-CAR from volatility and degradation, with a high thermal stability, and they also enabled the solubilization of the EO and *β*-CAR in water without the addition of an organic solvent. Therefore, it is possible to indicate the obtained products as potential candidates for commercial exploration since the ICs allow the complexed EO to exhibit a more stable chemical constitution than pure EO under storage conditions.

## 1. Introduction

The concern with food shortages in recent years resulted in a growing movement towards sustainable agriculture in order to preserve the environment, reduce costs, and increase productivity [1]. However, in large crops such as wheat and corn, invasive plants grow spontaneously and undesirably, causing a loss of crop productivity, jeopardizing the integrity of agricultural systems, and causing economic damage [2,3]. Continuous efforts have been made to protect crops from invasive plants, ranging from mechanical to chemical control [4]. The latter is the most used in modern agriculture, along with integrated management practices [5]. However, the excessive and inappropriate use of synthetic herbicides, such as glyphosate, to control weeds favors the selection of resistant genotypes, in addition to causing damage to the environment and animal and human health [6,7,8,9].

Thus, to overcome the disadvantages of synthetic herbicides, natural products obtained from plants are a viable, efficient, and safe alternative for farmers [10]. Essential oils (EOs) are secondary metabolism products of plants, play an important ecological role, and are biodegradable and widely available, as they can be obtained from plant leaves [11,12]. EOs may have herbicidal activity against weed species such as *Lolium multiflorum* (ryegrass) [13] and *Bidens pilosa* (beggarticks) [14].

The genus *Psidium* (Myrtaceae) contains species that produce EOs—generally rich in sesquiterpenes—which can inhibit seed germination and interfere with the development of different plant species [15]. Recently, the EO from the leaves of *Psidium gaudichaudianum* Proença & Faria (GAU), rich in *β*-caryophyllene, was reported for its phytotoxic properties on the root and aerial growth of *Lactuca sativa* L. (lettuce), presenting statistically similar results to the herbicide post-emergent glyphosate [16]. However, there are no reports in the literature concerning the herbicidal effect of *Psidium* EO species.

The GAU is an angiosperm species popularly known as araçá. Although the genus *Psidium* has a wide genetic diversity in an interspecific way, GAU is an under-examined native species [15]. Investigating understudied species is essential for their sustainable use.

The invasive plants *B. pilosa* and *L. multiflorum* are annuals and have different habitat preferences. *B. pilosa* (Asteraceae) originates from South America, and it is spread over all tropical and subtropical areas of the world with a hot and humid climate [17]. On the contrary, *L. multiflorum* (Poaceae) is a grass of European origin and commonly found in colder regions [18].

Although EOs have potential use for weed control, they are very volatile, and easily degraded when exposed to light, heat, and oxygen [19]. Thus, the use of protection methods that increase the thermal stability and water solubility of EOs is of interest to reduce such drawbacks and expand their applications. One of these methods utilizes 2-hydroxypropyl-*β*-cyclodextrin (HP*β*CD), an oligosaccharide derived from starch, to produce inclusion complexes (ICs) with EOs. HP*β*CD is soluble in aqueous media and has a nonpolar cavity that traps hydrophobic molecules, such as the compounds typically found in EOs [20].

To expand the knowledge concerning EOs, their potential applications, and to explore the possibility of creating a bioherbicide product, the GAU EO and its major compound *β*-caryophyllene were used in the present investigation. ICs of these materials with HP*β*CD were obtained, characterized by different analytical techniques, and had their effects against the weed species *L. multiflorum* and *B. pilosa* seeds evaluated. Furthermore, the cytogenotoxic action of the ICs in the cell cycle of root cells of *L. sativa* was verified. *L. sativa* was used because it is considered a model plant, as it has a low number of chromosomes (2*n* = 18) and these are relatively long (2.8 to 5.5 μm), which facilitates the microscopic visualization of cytogenetic changes, in addition to a high sensitivity [21,22].

## 2. Results and Discussion

**Figure 1:**
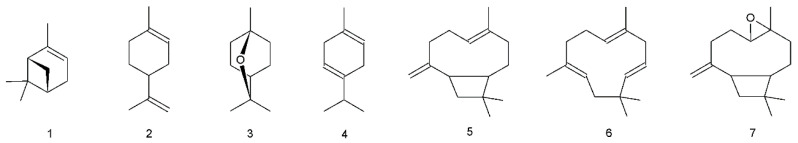
Structural formula of the compounds identified in the essential oil of the species *Psidium gaudichaudianum*. (**1**) *α*-pinene; (**2**) limonene; (**3**) 1,8-cineole; (**4**) *γ*-terpinene; (**5**) *β*-caryophyllene; (**6**) *α*-humulene; (**7**) caryophyllene oxide.

The EO from *P. gaudichaudianum* (GAU EO) showed an extraction yield of (0.58 ± 0.02)% (m m^−1^). The extraction yield of EO is important information to determine as it is directly correlated with its economic viability for large-scale exploration. To the best of our knowledge, it is not found in the literature yield of extraction for GAU EOs; thus, it is not possible to compare our findings with other results obtained from the same plant specimen. In fact, the most studied and registered *Psidium* EOs around the world are from the specimens *P. guajava*, *P. guineense*, and *P. cattleyanum*, with EO extraction yields of 0.3% [23], 0.1–0.9% [24], and 0.8% [25], respectively [15]. In general, EOs with commercial use can present an extraction yield between 0.30 and 3.86% (m m^−1^), which is the case for the EO obtained from GAU species [26]. When comparing the EO yield of GAU with the other species of the genus, it is observed that it can be considered a plant containing a quantity of EO with commercial interest. Regarding the density of the active compounds, GAU EO and *β*-caryophyllene densities were 0.9157 and 0.9005 g cm^−3^, respectively. The chemical composition of GAU EO and the chemical structures of the main components are shown, respectively, in Table 1 and Figure 1. Although the chromatogram contains about 100 compounds, only 7 of them were identified (A_relative_ > 2%), corresponding to 96.5% of the total composition of the EO. These compounds were grouped into terpene classes, being mono or sesquiterpenes, and may be hydrocarbons or oxygenated (Table 1). Among these, 70.3% were classified as sesquiterpenes and 26.2% as monoterpenes, being predominantly hydrocarbons. The major compounds (relative > 10%) present in the GAU EO were *β*-caryophyllene (53.3%) and *α*-humulene (12.0%). For the EO of the GAU species, Vasconcelos et al. (2019) [16] verified that the EO contained *β*-caryophyllene (17.0%) and *α*-humulene (5.5%), in addition to limonene (16.2%) and other minor compounds. This last work used the same plant as the present study. Despite the qualitative similarities in the compositions, quantitatively there were differences, which could be explained with environmental conditions, such as climatic factors and different periods of leaf collection.

The chemical profile of the EO from the leaves of GAU presented compounds similar to those reported for other species of the genus cultivated in other regions of the world [15]. Despite the variation in the composition of EOs in *Psidium* species, in general, it is observed that *β*-caryophyllene is a compound typically found in the genus. Considering that *β*-caryophyllene is the main compound found in the GAU EO, we decided to investigate the herbicidal and cytogenic activities of this terpene.

### 2.1. Preparation of Inclusion Complexes and Characterizations

The preparation method of the inclusion complexes was performed using maceration [23], a simple, highly efficient, and scalable method [27]. The guest (EO or *β*-caryophyllene) was mixed with the host (HP*β*CD) and after the addition of ethanol, a paste was formed, which was macerated, allowing the guest to be incorporated into the host cavities. To confirm the formation of inclusion complexes and determine the efficiency of the process, their characterization with analytical techniques is fundamental and these complement each other. This is possible due to variations in the physical properties of the guest molecule after inclusion complex formation. The analytical techniques utilized were spectroscopy in the UV-Vis region, GC, TGA, and FTIR.

**Figure 2:**
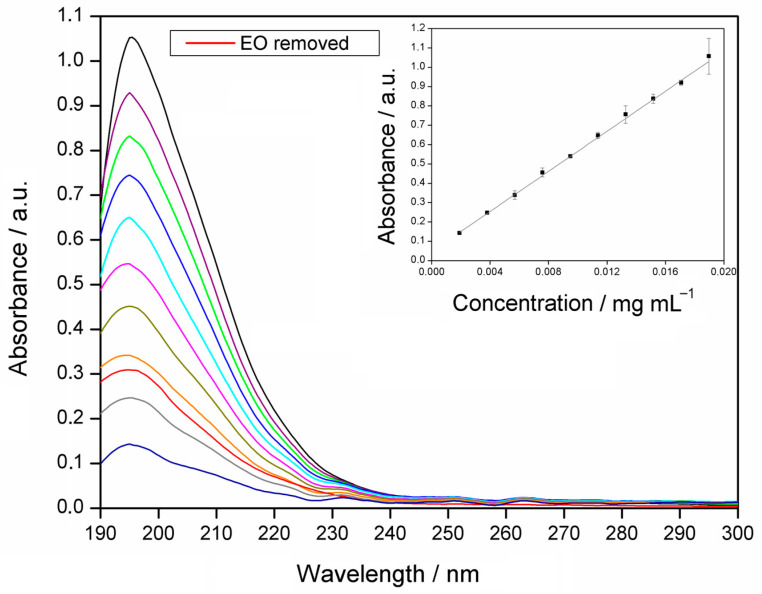
UV-Vis absorption spectrum of standard solutions of essential oil (EO) of *Psidium gaudichaudianum* in acetonitrile (1.9 to 19.0 μg mL^−1^) and EO removed from the inclusion complex. The insert is the analytical curve of EO in acetonitrile (λ_max_ = 195 nm).

The amount of EO complexed with HP*β*CD (complexation efficiency) was determined spectrophotometrically. For this purpose, the analytical curve of the EO of the GAU was obtained from a UV-Vis spectrophotometer (Figure 2). The maximum absorption wavelength occurs at 195 nm and this was adopted to identify and quantify the EO extracted from the IC. The analytical curve is shown as an insert in Figure 2 and the linear regression equation obtained was y = 51.453x + 0.0537, with R^2^_adjusted_ = 0.9913, and F_adjustment_ = 0.871 (*p* < 0.05). The complexation efficiency was (91.5 ± 0.6)%.

**Figure 3:**
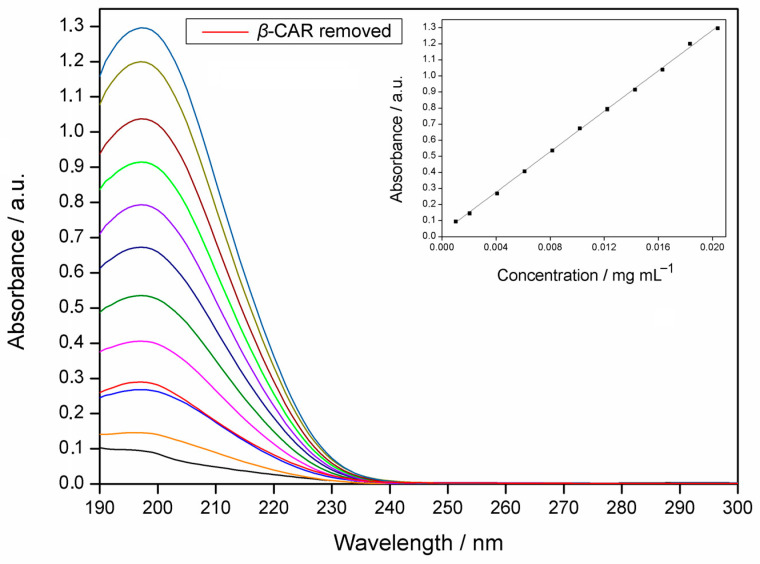
UV-Vis absorption spectrum of standard solutions of *β*-caryophyllene (*β*-CAR) in acetonitrile (1.0 to 20.4 μg mL^−1^), and *β*-CAR removed from the inclusion complex. As an insert is the analytical curve of *β*-CAR in acetonitrile (λ_max_ = 197 nm).

The verification of the *β*-caryophyllene complexation efficiency was performed by selecting the absorbance value at the wavelength of 197 nm (Figure 3). This was adopted to identify and quantify the *β*-caryophyllene extracted from the IC. The analytical curve is shown as an insert in Figure 3 and the linear regression equation obtained was y = 62.987x + 0.02119, with R^2^_adjusted_ = 0.9991, and F_adjustment_ = 0.000 (*p* < 0.05). The complexation efficiency was (83.9 ± 1.6)%.

The IC of GAU and *β*-caryophyllene achieved a complexation efficiency greater than 80%. Similar results, a complexation efficiency of 78% [28,29] and 97% [30], were achieved by other authors using maceration to obtain an inclusion complex of the analyte of interest with HP*β*CD. For the inclusion complex of linalool and HP*β*CD, using agitation in an aqueous solution as the method of preparation, the efficiency of the process was only 32% [31]. Therefore, the maceration process for the formation of inclusion complexes, containing more non-polar compounds, is useful.

**Figure 4:**
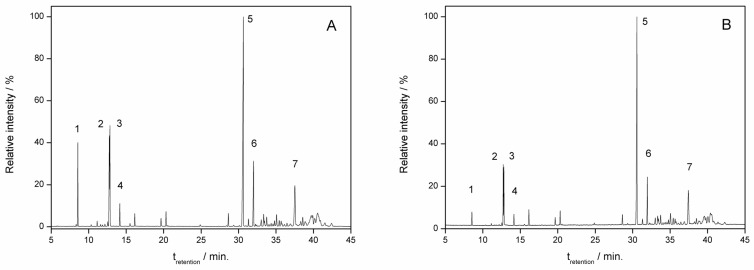
Essential oil chromatogram. (**A**) *P. gaudichaudianum*; (**B**) *P. gaudichaudianum* extracted from the inclusion complex. (**1**) *α*-pinene; (**2**) limonene; (**3**) 1,8-cineole; (**4**) *γ*-terpinene; (**5**) *β*-caryophyllene; (**6**) *α*-humulene; (**7**) caryophyllene oxide.

**Figure 5:**
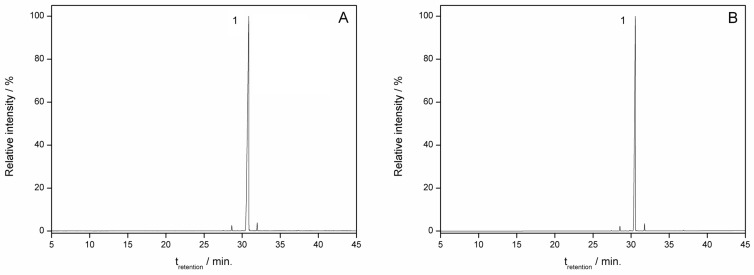
Chromatogram of (**A**) *β*-caryophyllene; (**B**) *β*-caryophyllene extracted from the inclusion complex. (**1**) *β*-caryophyllene.

The GAU EO and *β*-caryophyllene extracted from the corresponding ICs were characterized with chromatography under the same conditions as the non-complexed materials (Figure 4 and Figure 5). When comparing the chromatograms, it is observed that the major components of the EO are present in the IC (Figure 4) and *β*-caryophyllene was found in the IC (Figure 5). Thus, the similarity of the chromatograms of the non-complexed EO and the EO extracted from the IC qualitatively demonstrates the ability of HP*β*CD to trap hydrophobic compounds. From a qualitative point of view, other authors found similarities when comparing the chromatograms of EOs with EOs extracted from ICs [32].

**Figure 6:**
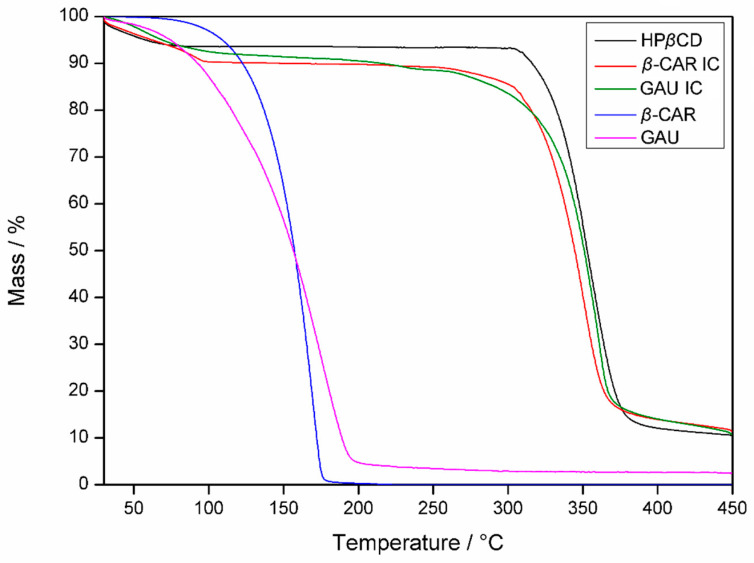
Thermogravimetric curves of HP*β*CD, *β*-caryophyllene (*β*-CAR), *Psidium gaudichaudianum* (GAU), and respective inclusion complexes (ICs).

Regarding the thermogravimetric analysis, the thermogravimetric curves are shown in Figure 6, and Table 2 contains the calculated mass losses for specific intervals of each sample. The TGA allows for checking the thermal stability of the samples with their mass variations, depending on the temperature, and its use is essential for confirming the formation of inclusion complexes.

The mass losses of the IC samples were divided into three regions: Δm_1_ refers to the loss of water molecules with the evaporation or volatilization of the EO from GAU and *β*-CAR (30–110 °C); Δm_2_ corresponds to the sample degradation or volatilization of EO and *β*-CAR or loss of water molecules that were retained in the HP*β*CD cavity (110–200 °C); and Δm_3_ is related to the degradation of the sample (200–400 °C).

Regarding HP*β*CD, initially there is a mass loss of 6.4% at temperatures below 110 °C. This is related to the release of adsorbed water in its structure, and up to 300 °C, the mass variation is only 0.1% when its decomposition has started. The third mass loss (Δm_3_) of HP*β*CD was the most evident and occurred with a maximum degradation at 351 °C. Thus, this oligosaccharide has a high thermal stability when compared to EO or *β*-CAR.

The EO of GAU showed a mass loss in a single event, with a maximum degradation or volatilization at 167 °C, and in Δm_2_, the mass loss was 77.9%. *β*-CAR had a gradual mass loss with the maximum degradation or volatilization content at 166 °C, with a Δm_2_ of 94.5%. Thus, EO and *β*-CAR show similar behavior and are completely degraded up to 200 °C. However, the GAU and *β*-CAR ICs show the most evident mass loss only in Δm_3_, being 76.5 and 75.9%, respectively. Therefore, ICs with HP*β*CD allow the thermal protection of the EO and *β*-CAR, as the maximum degradation occurs only at around 350 °C. In the Δm_2_ region, there was a small loss of mass in the ICs, confirming a different characteristic of the HP*β*CD, which practically did not lose mass in this temperature range. Therefore, this loss of IC mass may be associated with the volatilization of a small fraction of the EO and *β*-CAR that is adsorbed on the surface of the HP*β*CD. Thus, these may have been complexed on the external part of the HP*β*CD, which was also observed by Piletti et al. (2019) [33]. However, the Δm_2_ of EO and *β*-CAR is much higher than the values of ICs; therefore, samples complexed in the cyclodextrin cavity provide an increased thermal stability. Due to the ability of cyclodextrin to retain EO components to prevent volatilization, inclusion complexes can efficiently and stably complex guest molecules [34]. Thus, this advantage corroborates the hypothesis that EO and *β*-CAR were protected from thermal degradation, which is also reported by other authors [30,32,35,36].

Despite the IC curves being similar to HP*β*CD, between 200 and 300 °C, there was a loss of IC mass, which did not occur for the HP*β*CD curve (Figure 6). Therefore, this represents the loss of mass of the complexed samples, being evidence that complexation has occurred, as IC has different thermal properties from HP*β*CD. In another work, garlic EO, when complexed in *β*-cyclodextrin, presented changes in the thermal properties in this region of the thermogram, which was attributed to the complexation phenomenon [33,37]. Thus, these results indicate that the thermal properties were altered after the formation of the IC and these changes are a strong indication of the complexation of EO and *β*-CAR. Thus, the experiments with TGA corroborated the results of the complexation efficiency of the EO of the GAU and the *β*-CAR with HP*β*CD, which was not ideal (100%).

**Figure 7:**
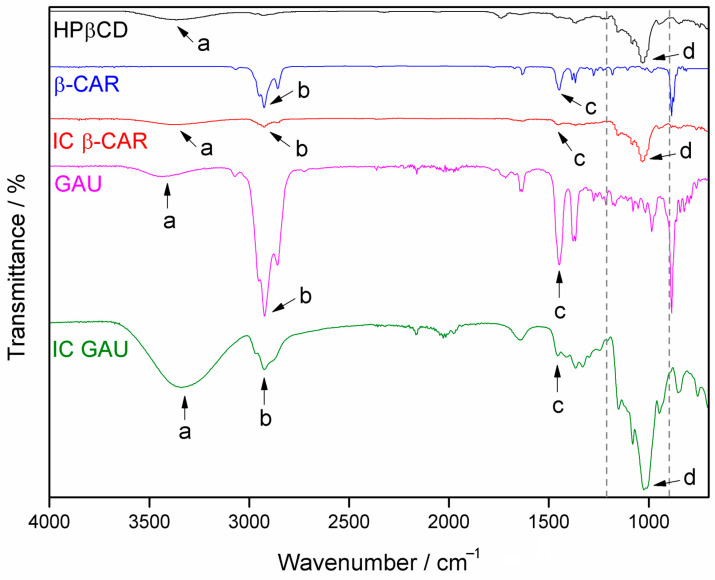
FTIR spectra for HP*β*CD, *β*-caryophyllene (*β*-CAR), *β*-CAR inclusion complex (IC), *Psidium gaudichaudianum* essential oil (GAU), and GAU IC.

An FTIR spectroscopy analysis is a widely used tool to provide qualitative information in identifying interactions between complexed molecules and HP*β*CD. Thus, it is important to observe bands present in the GAU EO and in the *β*-CAR that are in the ICs, as well as the HP*β*CD bands, indicating complexation. The obtained spectra are displayed in Figure 7. In the HP*β*CD spectrum, the **a** band around 3400 cm^−1^ refers to the stretching of the O-H bond, and the **d** band at 1030 cm^−1^ is associated with the stretching of the C-O bond of the ether moiety [38]. The **a** band is present in all spectra with the exception of *β*-CAR, as expected, since this terpene contains only carbon–carbon and carbon–hydrogen bonds. Similarly, the **d** band is only present in HP*β*CD and IC spectra.

The **b** band at 2925 cm^−1^, present in all spectra, refers to the C-H asymmetric stretching of the CH_2_ groups. The bands at 2860 and 2950 cm^−1^, observed mainly in the GAU and *β*-CAR spectra, belong to, respectively, the C-H symmetric and asymmetric stretching of the CH_3_ groups [38]. The **c** band at 1450 cm^−1^ is associated with the asymmetric angular deformation of CH_3_ groups and is mainly present in the spectra of GAU EO, *β*-CAR, and ICs [38].

Bands in the GAU spectrum with wavenumbers around 900 to 1200 cm^−1^ were suppressed in IC (between the dashed vertical lines). The disappearance of these bands in the IC spectrum may be related to the complexation of compounds with vibrations in this region, which were superimposed by bands present in the HP*β*CD [39,40]. This is because these bands were evident in the ICs simultaneously, even with a reduced size, confirming the presence of EO or *β*-CAR with HP*β*CD. Bands **a**, **b**, **c**, and **d** confirmed that there was interaction between the guests that are the EO and the *β*-CAR with the host that is the HP*β*CD. Other works also related the changes of the bands in the spectra as indicative of complexation [41,42].

### 2.2. Herbicidal Activity

The herbicidal potential of GAU EO, *β*-CAR, and the corresponding ICs was assessed at different concentrations against the weed species *B. pilosa* and *L. multiflorum*. The effect of the aforementioned materials on seed germination (G), root growth (RG), and aerial growth (AG) were evaluated. Glyphosate (PC1) and 2,4-D (PC2) were used as positive controls. The mixture of acetone (2%) and tween 80 (0.05%) in an aqueous medium was used as a negative control (NC) for GAU EO or *β*-CAR, and for ICs, HP*β*CD was used in an aqueous medium.

Initially, to verify the significance and compare the toxicities of EO, *β*-CAR, and their respective ICs, an analysis of variance was performed with all the evaluated results, which indicated that the treatments had a significant effect on seed germination, root growth, and shoot growth in *B. pilosa* and *L. multiflorum*, with a *p*-value of <0.001 (Table 3). Additionally, bidirectional and tridirectional interactions also showed significance, except for the interaction between species and concentration in the variables of the root and root growth. Therefore, we can compare the effect of the treatments on the weed plants and between them at the same concentrations regarding seed germination, root growth, and shoot growth (Table 4).

Comparing the response of each weed plant to the evaluated treatments, the results show that *L. multiflorum* was a more sensitive plant than *B. pilosa* (Table 4). This occurred because *L. multiflorum* had a lower percentage of germination and root growth compared to *B. pilosa* in most treatments and concentrations. Regarding shoot growth, the treatments had similar effects on the weed plants in most concentrations.

Furthermore, in general, it is noticeable that GAU EO is more toxic than all other treatments (Table 4). Thus, the synergistic effect of the EO is evidenced as the mixture of compounds is important for its biological application. Other studies have shown that EOs mainly composed of sesquiterpene compounds can contribute to phytotoxic activity [16,43]. It is also possible to establish a dose-dependent response for these plants, as G, RG, and AG decreased with increasing concentrations. However, the concentration of 375 µg mL^−1^ of *β*-CAR is an exception, as it led to the total inhibition of G, AG, and RG in *L. multiflorum* and AG in *B. pilosa* (Table 4).

**Figure 8:**
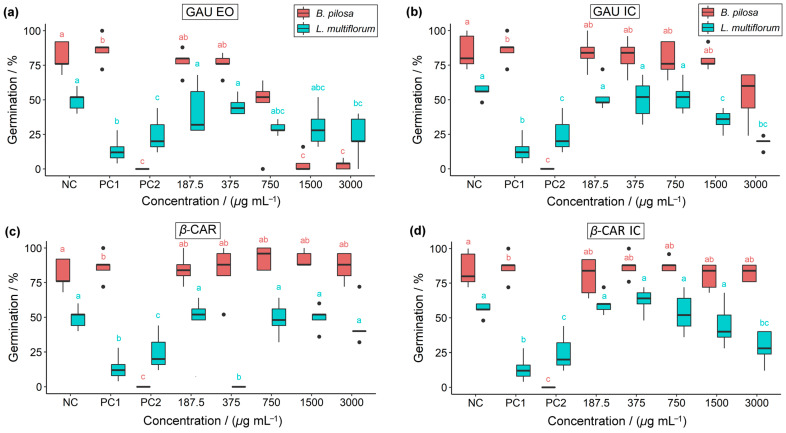
Herbicidal activity of (**a**) *P. gaudichaudianum* essential oil, (**b**) *P. gaudichaudianum* inclusion complex, (**c**) *β*-caryophyllene, and (**d**) *β*-caryophyllene inclusion complex on the germination of *B. pilosa* and *L. multiflorum*. NC = negative control; PC1 = positive control 1 (glyphosate); PC2 = positive control 2 (2,4-D). Boxplots followed by the same letters as the controls (NC, PC1, and PC2) do not differ significantly from each other using Dunnett’s test (*p* < 0.05).

**Figure 9:**
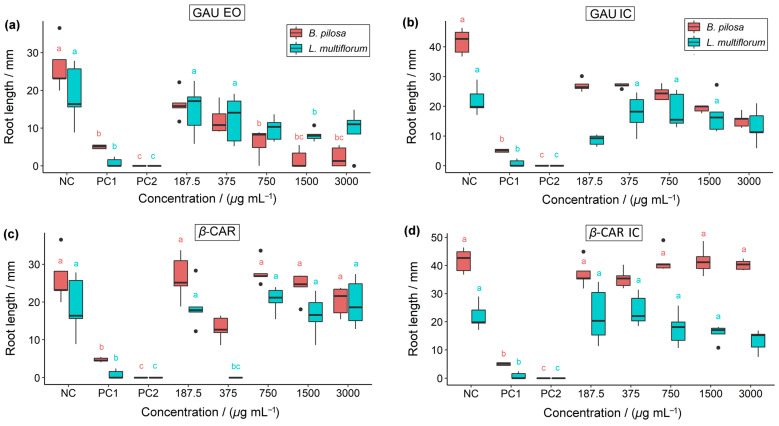
Herbicidal activity of (**a**) *P. gaudichaudianum* essential oil, (**b**) *P. gaudichaudianum* inclusion complex, (**c**) *β*-caryophyllene, and (**d**) *β*-caryophyllene inclusion complex on the root growth of *B. pilosa* and *L. multiflorum*. NC = negative control; PC1 = positive control 1 (glyphosate); PC2 = positive control 2 (2,4-D). Boxplots followed by the same letters as the controls (NC, PC1, and PC2) do not differ from each other according to Dunnett’s test (*p* < 0.05).

**Figure 10:**
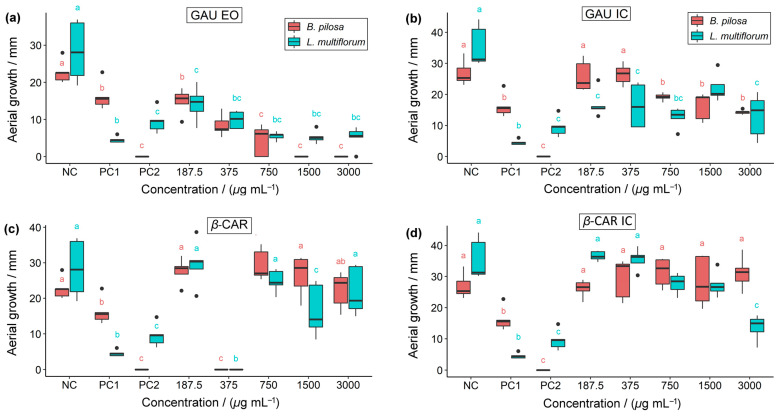
Herbicidal activity of (**a**) *P. gaudichaudianum* essential oil, (**b**) *P. gaudichaudianum* inclusion complex, (**c**) *β*-caryophyllene, and (**d**) *β*-caryophyllene inclusion complex on the aerial growth of *B. pilosa* and *L. multiflorum*. NC = negative control; PC1 = positive control 1 (glyphosate); PC2 = positive control 2 (2,4-D). Boxplots followed by the same letters as the controls (NC, PC1, and PC2) do not differ from each other according to Dunnett’s test (*p* < 0.05).

Seed germination, root growth, and shoot growth of the weed plants were assessed for all treatments and compared to the controls (Figure 8, Figure 9 and Figure 10).

The seed germination rate of the weed plants using the treatments was compared to the controls (Figure 8). Regarding GAU EO, it was observed that the germination in *B. pilosa* seeds, at higher concentrations (≥1500 µg mL^−1^), was similar to 2,4-D (PC2), where there was no germination. Glyphosate (PC1), being generally a post-emergence herbicide, does not affect the germination of *B. pilosa*. For GAU IC, only the concentration of 3000 µg mL^−1^ caused a reduction in the germination rate of *B. pilosa*, similar to what was observed at 750 µg mL^−1^ of GAU EO. For *B. pilosa*, *β*-CAR and *β*-CAR IC did not influence the germination variable, as they were similar to the negative control and glyphosate. Regarding *L. multiflorum* seeds, considering the use of GAU EO and GAU IC, the germination at 3000 µg mL^−1^ stands out for both, as they were similar to the positive controls. At a concentration of 375 µg mL^−1^, *β*-caryophyllene inhibited 100% of the germination of *L. multiflorum* seeds.

In general, most of the treatments used showed herbicidal activity, either by inhibiting seed germination or by affecting root and aerial growth. The GAU EO is a promising natural product as it exhibits herbicidal potential similar to glyphosate, a commercial product, especially at a concentration of 3000 µg mL^−1^ when evaluating RG and AG. Furthermore, it is observed that the inclusion complexes (ICs) exhibited herbicidal activity equal to or lower than the pristine GAU EO or *β*-CAR. Despite this, the use of inclusion complexes instead of the EO allows for expanding their applications in the agricultural sector as the ICs improve their properties, as discussed earlier. Therefore, it is recommended to use the GAU IC at a concentration of 3000 µg mL^−1^ for *B. pilosa* and *L. multiflorum*, as it showed a moderate negative effect on G, AG, and RG. As for the *β*-CAR IC, the recommended concentration for use in *L. multiflorum* is 3000 µg mL^−1^, as it exhibits a greater toxicity.

Regarding root growth, both GAU EO and GAU IC in *B. pilosa* showed a lower growth compared to the negative control (Figure 9). However, GAU EO exhibited root growth inhibition similar to glyphosate at concentrations ≥ 750 µg mL^−1^. As for *β*-caryophyllene and *β*-CAR IC, only the concentration of 375 µg mL^−1^ inhibited root elongation in *B. pilosa*, without resembling any of the controls. For *L. multiflorum*, notable results were observed with GAU EO at a concentration of 1500 µg mL^−1^, where RG was similar to glyphosate. With GAU IC, a decrease in RG was observed at concentrations of 187.5 and 3000 µg mL^−1^, indicating a higher toxicity. At a concentration of 375 µg mL^−1^, *β*-CAR completely inhibited root growth in *L. multiflorum*. As for *β*-CAR IC, 3000 µg mL^−1^ was the concentration that most inhibited RG in *L. multiflorum*.

For the variable of aerial growth in *B. pilosa*, in the GAU EO, concentrations ≥ 750 µg mL^−1^ stand out, as there was no development of the aerial part of the plant, resembling the effects of 2,4-D (Figure 10). The IC also showed the highest inhibition values for AG with concentrations ≥ 750 µg mL^−1^, resembling the effects of glyphosate. The AG of *B. pilosa* and *L. multiflorum* was completely inhibited using 375 µg mL^−1^ of *β*-CAR. The concentration of 3000 µg mL^−1^ of the *β*-CAR IC had the greatest impact on the AG of *L. multiflorum*. All concentrations of the GAU EO and the GAU IC affected the AG of *L. multiflorum*.

The evaluation of herbicidal properties in invasive plants using GAU EO and *β*-CAR has not been previously reported in the literature. However, this EO has been tested on model plants such as lettuce and sorghum [16]. In that study, GAU EO affected the root growth of lettuce at concentrations ≥ 750 µg mL^−1^, similar to glyphosate, which is consistent with the observations regarding invasive plants [16]. Another study that used inclusion complexes of nine EOs in HP*β*CD found that there was no improvement in the toxicity on lettuce (*L. sativa*) and ryegrass (*L. perenne*) compared to the non-complexed EOs [3].

The strategy to produce a nanoemulsion containing sweet fennel EO (*Foeniculum vulgare*) was used to protect the bioactive compounds present in the EO and maintain its herbicidal activity on invasive plants of wheat (*Triticum aestivum*) [1]. Although the effectiveness of the nanoemulsion was not compared to the EO itself, the nanoemulsion showed the ability to inhibit G, RG, and AG [1], corroborating with our findings.

Ideally, the EO or EO IC should be toxic to invasive plants without affecting agricultural crops, as the herbicide would be applied in an environment where these plants coexist. In this context, M. Ibáñez and Blázquez (2019) [13] used EOs predominantly composed of sesquiterpene compounds and found promising herbicidal activity in *L. multiflorum* and other invasive plants [44]. This study used the same concentrations of EOs on non-target plants (tomato, cucumber, and rice), and no significant phytotoxic effects were observed [44].

### 2.3. Cytogenotoxic Activity

The analysis of cytogenotoxicity was performed to assess the toxic effect of the essential oil of *P. gaudichaudianum*, *β*-caryophyllene, and their respective inclusion complexes on the cell cycle of the model plant *L. sativa*, thereby determining the mode of action involved in the inhibition of germination and root growth. Table 5 and Table 6 present the results of the induced alterations on the mitotic index and the observed genotoxic and mutagenic potential.

**Figure 11:**
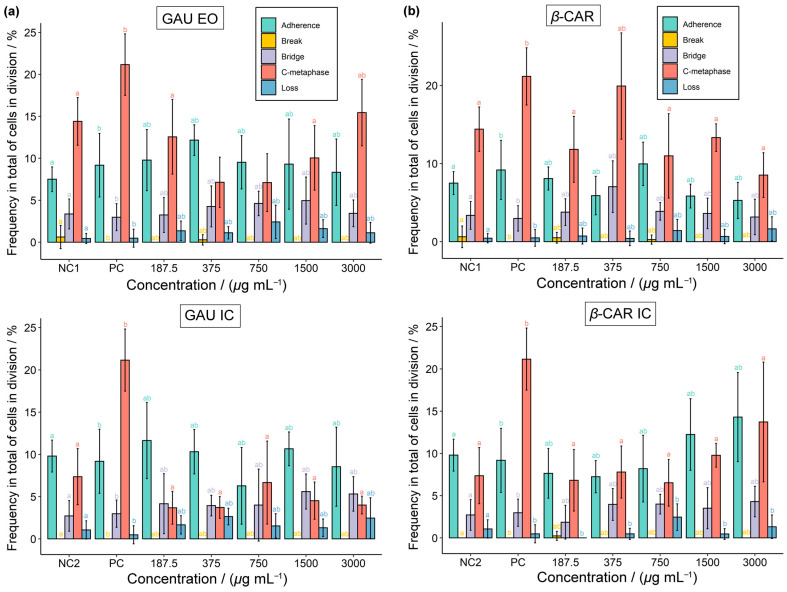
Percentage of chromosomal abnormalities observed in meristematic cells of *Lactuca sativa* roots exposed to (**a**) essential oil of *Psidium gaudichadianum* (GAU EO) and its inclusion complex (GAU IC) and (**b**) *β*-caryophyllene (*β*-CAR) and its inclusion complex (*β*-CAR IC). NC1 = negative control 1 (acetone and tween 80 in aqueous medium); NC2 = negative control 2 (HP*β*CD in aqueous medium); PC = positive control (methyl methanesulfonate). Means that share different letters between lines differ statistically from each other with Dunnett’s test (*p* < 0.05).

**Figure 12:**
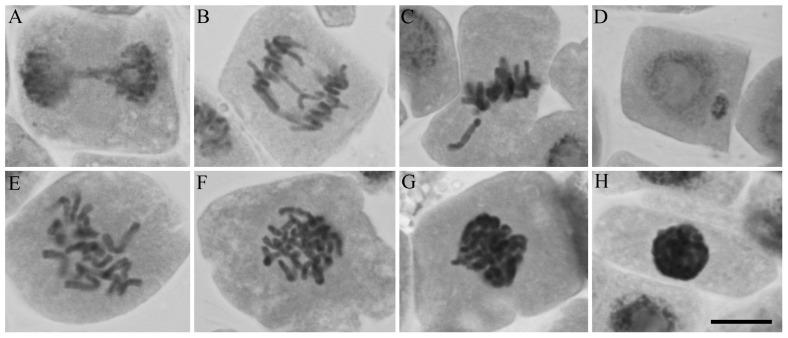
Chromosomal and nuclear abnormalities observed in meristematic cells of *Lactuca sativa* exposed to the EO, *β*-CAR, and their respective ICs. (**A**) Telophase bridge; (**B**) Anaphase with bridge and lost chromosome; (**C**) Metaphase with lost chromosome; (**D**) Interphase with micronucleus; (**E**,**F**) C-metaphase; (**G**) Chromosome adherence; (**H**) Condensed nucleus. Scale bar = 10 μm.

The percentage of each chromosomal alteration within the total number of dividing cells was evaluated (Figure 11). The images of chromosomal and nuclear abnormalities observed under the microscope in meristematic cells of *Lactuca sativa* exposed to the EO, *β*-CAR, and their respective inclusion complexes are presented in Figure 12. Despite the controls showing little difference in the mitotic index (Table 5 and Table 6), the positive control exhibited a higher genotoxicity, with a greater number of chromosomal alterations, especially in the c-metaphase (Figure 11).

Cytotoxicity was estimated through the analysis of the mitotic index. The mitotic index is related to cells undergoing division, and its decrease is caused by the disruption of one or more mitotic phases or by a slowdown in the cell division rate of the root apical meristem [45]. The reduction in cell division results in decreased root elongation [46]. Chromosomal and nuclear abnormalities (genotoxic effect) also lead to changes in the mitotic index as they interfere with cell viability [47]. Thus, cytotoxic compounds can also demonstrate genotoxicity [47,48].

Genotoxic effects can be caused by aneugenic and clastogenic mechanisms of action. Aneugenic agents induce errors in the attachment of chromosomes to the mitotic spindle, resulting in chromosomal abnormalities such as the c-metaphase, chromosome losses, and chromosomal adhesions. Clastogenic agents directly interact with DNA, causing chromosomal breaks and bridges [21,49].

Mutagenicity was assessed using the presence of cells with a micronucleus. This nuclear abnormality is a marker of toxicity. A micronucleus can originate from chromosomal breaks caused by clastogenic agents or from chromosomal losses caused by aneugenic agents [49,50,51].

In relation to the mitotic index, the EO GAU and *β*-CAR showed similarities to the controls, except for the concentration of 375 µg mL^−1^ of *β*-CAR (Table 5). For this concentration, the complete inhibition of G, RG, and AG in *L. multiflorum* was observed (Table 4). Thus, the cytotoxic effect of *β*-CAR justifies these herbicidal alterations at a macroscopic level. If the mitotic index is low, it means that a smaller number of cells are dividing, making it more difficult to find chromosomal abnormalities (genotoxicity).

At a concentration of 375 µg mL^−1^, the genotoxicity of *β*-CAR, despite not being greatly affected and resembling the controls (Table 5), showed the highest frequency of chromosomal abnormalities (Figure 11b), especially in terms of the c-metaphase and bridge formations (Figure 12A,E,F), compared to other concentrations. Thus, the mechanisms of action of *β*-CAR involve clastogenic and aneugenic effects. At concentrations of 1500 and 3000 µg mL^−1^, the genotoxicity of *β*-CAR was less affected than at other concentrations. Regarding mutagenicity, although the GAU EO, *β*-CAR, and inclusion complexes resembled the controls, with no presence of a condensed nucleus or micronucleus (Figure 12H), they were observed in the treatments, such as at 375 µg mL^−1^ of *β*-CAR, indicating a potential mutagenic effect (Table 5 and Table 6).

The GAU EO showed the c-metaphase and chromosome adherence (Figure 12E–G) as the main chromosomal abnormalities (Figure 11a). Thus, the primary mechanism of action of the EO was attributed to aneugenic agents. At a concentration of 375 µg mL^−1^ of the GAU EO, chromosomal breaks were observed (Figure 11a). Additionally, the EO exhibited the presence of a condensed nucleus or micronucleus at 375, 1500, and 3000 µg mL^−1^ (Table 5).

The GAU IC exhibited a higher cytotoxicity at concentrations equal to or above 750 µg mL^−1^, as indicated by the lower mitotic indices (Table 6). If the cell cycle is affected, abnormal cell proliferation can contribute to decreased germination and root and aerial growth. This was observed in the herbicidal activity, as higher concentrations of GAU IC had a greater impact on G, RG, and AG. Although GAU IC did not affect genotoxicity, the main chromosomal abnormalities observed were the c-metaphase, chromosome adherence, and bridge (Figure 11a). Therefore, the mechanisms of action of GAU IC were clastogenic and aneugenic. At 375 and 3000 µg mL^−1^, there was evidence of mutagenicity, as micronuclei were observed when using GAU IC (Table 6).

The *β*-CAR IC exhibited a mitotic index and genotoxicity similar to the positive control at 187.5 and 3000 µg mL^−1^, respectively. The main chromosomal abnormalities observed were the c-metaphase and chromosome adherence, indicating an aneugenic effect (Figure 11b). At 375 µg mL^−1^, the *β*-CAR IC showed evidence of mutagenicity.

In general, the least observed chromosomal abnormalities among the treatments were breakage and loss (Figure 11). The most observed abnormalities were the c-metaphase and chromosome adherence. The c-metaphase is associated with spindle malfunction or inactivation, halting the mitotic cycle at the metaphase [49,52]. Therefore, the presence of the c-metaphase indicates that the spindle is likely being damaged by proteases contained in the GAU EO or *β*-CAR [53]. Chromosome adherence leads to the loss of normal condensation characteristics and the formation of clusters, and it is related to cytotoxic effects [54,55].

In essential oils from *Psidium*, chromosomal abnormalities, mainly of the c-metaphase and chromosome adherence types, have also been observed in *L. sativa* [16]. Thus, the results obtained in this study are similar to those of Vasconcelos et al. (2019) [16], who used the GAU EO and observed that compounds with cytotoxic effects induced chromosomal abnormalities in *L. sativa*, indicating genotoxic effects. According to Nishida et al. (2005) [56], in general, essential oils, including the compounds 1,8-cineole and *α*-pinene present in the GAU EO, have the ability to inhibit DNA synthesis, preventing the cell from entering mitosis and thus exerting an aneugenic effect.

It was possible to verify that the GAU EO, *β*-CAR, and their respective ICs interfere with the cell cycle of *Lactuca sativa* roots and induce chromosomal abnormalities. The results allowed for establishing a correlation between microscopic and macroscopic parameters, justifying the demonstrated phytotoxicity.

## 3. Materials and Methods

### 3.1. Standards and Reagents

The reagents *β*-caryophyllene (96.2%, Quinarí, lot CAR76HG, Ponta Grossa, PR, Brazil), 2-hydroxypropyl-beta-cyclodextrin (HP*β*CD, Oakwood Chemical, Estill, SC, USA), ethanol (95%, Didática, São Paulo, SP, Brazil), acetonitrile (99.9%, Sigma-Aldrich, St. Louis, MO, USA), acetone (99.5%, Dinâmica, Indaiatuba, SP, Brazil), tween 80 (Êxodo Científica, Hortolândia, SP, Brazil), glyphosate (Roundup, São José dos Campos, SP, Brazil), 2,4-dichlorophenoxyacetic acid (2,4-D) (98%, Sigma-Aldrich, St. Louis, MO, USA), hydrochloric acid (37%, Neon, Suzano, SP, Brazil), methyl methanesulfonate (MMS) (99%, Sigma-Aldrich, St. Louis, MO, USA), aceto-orcein (Dinâmica, Indaiatuba, SP, Brazil), and acetic acid (99.7%, Vetec, Rio de Janeiro, RJ, Brazil) were used as received. To prepare the solutions, type 1 water obtained from a PURELAB system (PURELAB Ultra MK2) was utilized.

### 3.2. Collection and Extraction of Essential Oil from the Leaves of P. gaudichaudianum

*Psidium gaudichaudianum* plants are located at the Center for Agricultural Sciences and Engineering at the Federal University of Espírito Santo (CCAE/UFES). Leaf samples of plants were collected in July 2021 at 8 am in Alegre, Espírito Santo, Brazil, located at south latitude (20°45′), west longitude (41°31′), and an altitude of 254 m.

A sample of *P. gaudichaudianum* was collected, dried, and deposited in the RB herbarium of the Botanical Garden of Rio de Janeiro (RB00774107). Leaf samples were collected around the canopy of trees. The material was stored in paper bags and the leaves were dried in the shade at room temperature (~25 °C) for about a week. Then, the leaves were stored in a freezer at −9 °C until EO extraction.

The essential oil was extracted using hydrodistillation for a period of 4 h, following the methodology recommended by the Brazilian Pharmacopoeia for the extraction of volatile oils [57]. In the extractions, carried out several times, about 100 g of dry leaves were used in approximately 1500 mL of type 1 water in a 2000 mL round-bottom flask. The EO was extracted and subsequently stored in a freezer at −20 °C, protected from light until its use and/or characterization.

### 3.3. Determination of Absolute Density and Yield of Essential Oil Extraction

The absolute density of GAU EO and *β*-caryophyllene was determined using a 5 mL pycnometer (RBR Vidros, São Paulo, Brazil). The masses of the empty and dry pycnometer, containing water and GAU EO, were weighed. Density calculation was performed using Equation (1).

(1) $$d=\frac{m_{2}-m_{1}}{m_{3}\;-\;m_{1}}\;\times \;(d_{\mathrm{water}}\;-\;d_{\mathrm{air}})\;+\;d_{\mathrm{air}}$$

where d = the absolute density of the GAU EO (g cm^−3^); m_1_ = dry and empty pycnometer mass (g); m_2_ = pycnometer mass with the GAU EO (g); m_3_ = the mass of the pycnometer with water type 1 (g); d_water_ = the density of water at 25 °C (9.97 × 10^−1^ g cm^−3^); and d_air_ = air density (1.18 × 10^−1^ g cm^−3^).

The GAU EO extraction yield was determined, in triplicate, with the ratio between the extracted EO mass and the dry plant mass multiplied by 100.

### 3.4. Essential Oil Chromatographic Profile

Gas chromatography with flame ionization detection (GC-FID, Shimadzu QP2010SE, Kyoto, Japan) and gas chromatography coupled with mass spectrometry (GC-MS, Shimadzu QP2010SE, Japan) were used to identify and semi-quantify the constituents of the essential oil from *P. gaudichaudianum* leaves and *β*-caryophyllene. The analysis method employed was based on the methodology described by Mendes et al. (2017) [58] and Souza et al. (2017) [59] using helium gas as a carrier gas for both detectors. Briefly, the injected volume consisted of 1 μL of an essential oil solution with a concentration of 3% in 99.9% acetonitrile. The injector temperature was maintained at 220 °C with a split ratio of 1:30. A fused silica capillary column with dimensions of 30 m × 0.25 mm and a stationary phase of Rtx^®^-5MS with a film thickness of 0.25 μm were used. The oven temperature was initially programmed at 40 °C for 3 min, followed by a gradual increase of 3 °C min^−1^ until reaching 180 °C, where it was held for 10 min. The temperatures of the FID and MS detectors were set at 240 °C and 200 °C, respectively.

The GC-MS analyses were conducted using electron impact ionization equipment with an energy of 70 eV. As for the GC-FID analyses, they were performed using a flame formed by a mixture of H_2_ and atmospheric air at a temperature of 300 °C.

The identification of the constituents of the GAU EO and *β*-caryophyllene was performed by comparing the mass spectra obtained with those available in spectral databases. Additionally, the retention index (RI) was used to assist in the identification. To calculate the RI, a mixture of saturated alkanes C_7_–C_40_ was used, following the same chromatographic conditions as the GAU EO and *β*-caryophyllene. The adjusted retention time for each compound was obtained through gas chromatography with flame ionization detection (GC-FID). Then, the calculated values for each compound were compared with the values found in the literature [60,61,62].

The relative percentage of each compound present in the GAU EO and *β*-caryophyllene was determined by calculating the ratio between the integrated peak area and the total area of all constituents in the sample, provided that their relative area was above 2%.

### 3.5. Preparation of the Inclusion Complex

The ICs were obtained with the maceration method following the methodology of Mendes et al. (2023) [63]. ICs containing EO were prepared as follows: EO and HP*β*CD were mixed in a molar ratio of 1:1 and a total maceration time of 35 min was used. About 3.9 g of HP*β*CD (1460 g mol^−1^ = 0.00267 mol) and 0.5 g of EO (187.47 g mol^−1^ = 0.00267 mol) were weighed in a mortar. The average molar mass was obtained considering the molar mass of each compound and their respective percentages of relative area obtained from the chromatographic analysis of the EO. The experiments were performed in triplicate. The mixture was manually macerated for 5 min using a mortar and pestle. Then, the volume of 95% ethanol corresponding to 2 mmol mL^−1^ of HP*β*CD was added to form a homogeneous paste and then the mixture was macerated for 30 min. Similarly, the IC containing *β*-caryophyllene was prepared in triplicate, varying only the amounts of HP*β*CD and *β*-caryophyllene (204.36 g mol^−1^), which were 3.6 and 0.5 g, respectively. The ICs produced were kept at 5 °C in a vacuum desiccator for 72 h. Subsequently, ICs were stored in amber bottles at −20 °C.

### 3.6. Characterization of the Inclusion Complex

#### 3.6.1. Analytical Curve

To determine complexation efficiency (CE), an analytical curve of EO and *β*-caryophyllene was obtained using a NanoDrop UV-Vis spectrophotometer (Thermo Scientific, Nanodrop One, Waltham, MA, USA), with a 0.5 nm resolution and quartz cuvettes with 1 cm of an optical path. Analyzes were performed in the scan mode in the range of 190 to 850 nm.

For this analysis, ten standard solutions were prepared in triplicate with increasing concentrations of EO and *β*-caryophyllene in acetonitrile, namely 1.9 to 19.0 μg mL^−1^ and 1.0 to 20 4 μg mL^−1^, respectively. The absorbance values at 195 and 197 nm, for the GAU EO and *β*-caryophyllene, respectively, were obtained for the construction of the curve, whose points were submitted to linear regression with the Least Squares Method.

To quantify the concentration of EO and *β*-caryophyllene removed from ICs, 25 mg of the materials was dissolved in 50 mL of 99.9% acetonitrile, and the system was kept in the dark at 900 rpm on a magnetic stirrer (Thermo Scientific, Cimerac, China) for 48 h. This procedure was performed in triplicate and adapted from Hill et al. (2013) [64]. Subsequently, the samples were centrifuged in a centrifuge (206-BL, Fanem, São Paulo, Brazil) at 1900 rpm for 20 min at 25 °C to decant the HP*β*CD, leaving only the EO or *β*-caryophyllene as soluble in acetonitrile. The solutions containing EO and *β*-caryophyllene in the solvent were submitted to the UV-Vis analysis, monitoring at 195 and 197 nm, respectively. The concentrations of EO and *β*-caryophyllene were obtained with the analytical curve, allowing the determination of the extracted EO or *β*-caryophyllene masses. Complexation efficiencies were determined using Equation (2):

(2) $$\mathrm{CE}\;(\%)=\frac{m_{\mathrm{ext}}\left(\mathrm{mg}\right)}{m_{\mathrm{add}}\;(\mathrm{mg})}\;\times \;100$$

where m_ext_ is the mass of the EO or *β*-caryophyllene extracted from the IC (mg) and m_add_ is the mass of the EO or *β*-caryophyllene added at the beginning of the complexation process (mg).

#### 3.6.2. Thermogravimetric Analysis

Thermal stability was measured using the thermogravimetric analysis (TGA) with the aid of a DTG thermal analyzer (60H, Shimadzu, Kyoto, Japan). Approximately 4 mg of HP*β*CD, EO, *β*-caryophyllene and ICs were weighed on an analytical microbalance (Mettler Toledo, XP26, São Paulo, Brazil). Each sample was individually transferred to an alumina crucible and subjected to heating in the temperature range of 30 to 450 °C, with a heating rate of 10 °C min^−1^. An inert atmosphere of nitrogen was used with a flow rate of 50 mL min^−1^.

#### 3.6.3. Fourier Transform Infrared Spectroscopy

The HP*β*CD, EO, *β*-caryophyllene, and ICs were submitted to a Fourier transform infrared spectroscopy (FTIR) analysis. An attenuated total reflectance (ATR) spectrophotometer with germanium crystal (Nicolet 6700, Thermo Scientific, Waltham, MA, USA) was used in the region of 4000 to 700 cm^−1^, with 32 scans and a spectral resolution of 4 cm^−1^.

### 3.7. Herbicidal Activity Evaluation

EO and *β*-caryophyllene emulsions were prepared using acetone (2% v v^−1^) and tween 80 (0.05% v v^−1^) in an aqueous solution, presenting the following concentrations: 3000, 1500, 750, 375, and 187.5 μg mL^−1^. IC solutions were prepared at the same concentrations, however, only with water. Negative controls were acetone (2% v v^−1^) with tween 80 (0.05% v v^−1^) and HP*β*CD in water. The positive controls were glyphosate (1 mL L^−1^) and 2,4-D (3 mmol L^−1^).

For the germination rate, root growth (RG) and aerial growth (AG) evaluations involved 3 mL of the solution of each treatment (including controls) being added to Petri dishes (9 cm in diameter) containing *B. pilosa* and *L. multiflorum* seeds on filter paper. The treatments were performed with 25 seeds and five replications. The plates were sealed with plastic film and kept in a germination chamber (BOD) at (24 ± 2) °C with a photoperiod of 16 h in light and 8 h in the dark. The test was carried out with a completely randomized design.

The percentage of germination (%G) and the RG and AG of *B. pilosa* and *L. multiflorum* were evaluated 7 days after the initial exposure of the seeds to the treatments. Air and root growth was measured with a digital caliper (Stainless Hardened, India).

### 3.8. Cytotoxicity Analysis

The cytogenetic evaluation was performed on the *L. sativa* model plant to visualize mitotic changes in its cell cycle, such as chromosomal abnormalities and involving a micronucleus [22]. This species has seeds with rapid and uniform germination, in addition to being sensitive to the action of toxic agents [65].

To carry out the experiments, 2 mL of the solution of each treatment of the emulsion and the inclusion complex of EO and *β*-caryophyllene (including negative controls) was added to Petri dishes (9 cm in diameter) containing 25 seeds of *L. sativa* on filter paper. Methyl methanesulfonate (MMS) (4 mmol L) was used as a positive control, once it was a DNA alkylating agent in cytogenotoxic tests [66]. After 48 h of exposure to seed treatments, 10 roots from each replication were collected and fixed in an ethanol/acetic acid (3:1 v v^−1^) solution and stored at −18 °C.

For the microscopic evaluation, slides with root meristematic cells were prepared. Before fixing the roots, they were washed with distilled water and subjected to hydrolysis in 5 mol L^−1^ of hydrochloric acid (HCl) for a period of 18 min at room temperature. The slides were prepared using the compression technique and stained with aceto-orcein (2% m v^−1^). Then, they were covered with a coverslip, sealed, and stored in a cold and humid chamber for the analysis and conservation of the material. One thousand cells per slide were evaluated, totaling five thousand cells per concentration.

The evaluation of cytotoxicity was performed by calculating the ratio between the total number of dividing cells and the total number of cells evaluated, using the mitotic index (MI). Genotoxicity was determined using the sum of the frequencies of cells with chromosomal (chromosomal abnormalities—CA) (non-oriented chromosomes, adherent chromosomes, c-metaphases, bridges, and chromosomal breaks) and nuclear abnormalities (NA) (nuclear communication, nuclear bud, multinucleated cells, and condensed nucleus) obtained using the ratio between the total number of cells with alteration and the total number of evaluated cells. Mutagenicity was determined using the frequency of cells with a micronucleus obtained using the ratio between the total number of cells with a micronucleus and the total number of evaluated cells [67]. The percentage of each chromosomal alteration within the total number of dividing cells was also evaluated.

### 3.9. Statistical Analysis

Linear regression was performed using a Minitab program, version 17. Phytotoxicity experiments (%G, RG, and AG) were performed as a three-way factorial. The weed species *B. pilosa* and *L. multiflorum* were the first factors. The second factor included four treatments (GAU EO, GAU IC, *β*-CAR, and *β*-CAR IC), and the third factor consisted of the five treatment concentrations (3000, 1500, 750, 375, and 187.5 µg mL^−1^). For phytotoxic and cytotoxic assays (MI, NA, and CA), data were submitted to the ANOVA (analysis of variance) with a randomized design. The statistical comparison of data between factors was performed using Tukey’s test and comparison with controls involved Dunnett’s test at 5% significance, using R Studio software version 4.1.0 [68].

## 4. Conclusions

The use of natural products such as *Psidium gaudichaudianum* essential oil, the major compound *β*-caryophyllene, and their inclusion complexes shows promising effects in weed management in agricultural fields. Since essential oils are considered environmentally safe and easily degradable in nature, they can be an alternative to reduce the use of commonly employed herbicides. The produced inclusion complexes were able to protect the EO and *β*-CAR from volatility and thermal degradation, with a high complexation efficiency. The comparison between the mean effects of treatments with *P. gaudichaudianum* essential oil, *β*-caryophyllene, and the respective inclusion complexes and their concentrations on germination and root and aerial growth in *B. pilosa* and *L. multiflorum* was studied. The IC of GAU EO at the highest concentration tested showed a significant effect against the weed species *B. pilosa* and *L. multiflorum*. However, the *β*-CAR IC at the highest concentration was effective only in *L. multiflorum*. The inclusion complexes maintained or decreased the biological activity against weed plants compared to pristine GAU EO and *β*-CAR, but positively, the IC enables the EO application in the field, as it protects the EO from volatilization while enhancing its solubilization in water. However, the advantages of using the ICs outweigh this factor by improving the properties of the essential oil and allowing its field application. Future studies focusing on the toxicity of these inclusion complexes in crops such as wheat and corn are necessary. Nevertheless, the advances achieved in this work provide a commercially viable product for weed control.

## Figures and Tables

**Table 1:** Chemical composition of the essential oil of the species *Psidium gaudichaudianum*.

*n*	Compound ^a^	t_retention_ ^b^	A_relative_ (%) ^c^	Molar Mass (g mol^−1^)	Terpene Classification ^d^
**1**	*α*-pinene	8.549	9.2	136.24	HM
**2**	limonene	12.833	9.8	136.24	HM
**3**	1,8-cineole	12.833	4.3	154.25	OM
**4**	*γ*-terpinene	14.149	2.9	136.24	HM
**5**	*β*-caryophyllene	30.646	53.3	204.36	HS
**6**	*α*-humulene	32.003	12.0	204.36	HS
**7**	caryophyllene oxide	37.519	5.0	220.36	OS
	Total identified		96.5		

^a^ Major compounds listed in order of elution using Rtx^®^-5MS column. ^b^ Retention time. ^c^ Compounds with relative area > 2% were identified. ^d^ Terpene classification: hydrocarbon monoterpene (HM), oxygenated monoterpene (OM), hydrocarbon sesquiterpene (HS), and oxygenated sesquiterpene (OS).

**Table 2:** Weight loss of HP*β*CD, *Psidium gaudichaudianum* (GAU), *β*-caryophyllene (*β*-CAR), and respective inclusion complexes (ICs).

Sample	Δm_1_ (%) ^a^ 30–110 °C	Δm_2_ (%) ^b^ 110–200 °C	Δm_3_ (%) ^c^ 200–400 °C
HP*β*CD	6.4	0.1	81.4
GAU	17.4	77.9	-
*β*-CAR	5.3	94.4	-
GAU IC	7.9	1.6	76.5
*β*-CAR IC	9.8	0.4	75.9

Mass variation: ^a^ loss of water or volatilization, ^b^ degradation, volatilization, or loss of water that was retained in the HP*β*CD cavity, and ^c^ sample degradation.

**Table 3:** Analysis of variance for the effect of *P. gaudichaudianum* essential oil, *β*-caryophyllene, and the respective inclusion complexes on germination and root and aerial growth of *Bidens pilosa* and *Lolium multiflorum*.

Source of Variation	*df*	Germination (%)	Root Growth (mm)	Aerial Growth (mm)
Invasive plant species (A)	1	345.2801 ***	160.437 ***	4.9259 *
Treatment (B)	3	63.6608 ***	158.2757 ***	219.206 ***
Concentration (C)	4	26.706 ***	8.5239 ***	26.9576 ***
A X B	3	27.9444 ***	58.5316 ***	5.4023 **
A X C	4	3.6755 **	1.5868	2.1215
B X C	12	13.4208 ***	10.8153 ***	24.6083 ***
A X B X C	12	7.519 ***	4.804 ***	7.7438 ***
Coefficient of variation		20.53%	23.69%	23.37%

*df* Degrees of freedom and number of replications (*n*): 5. * Significant at 5% probability levels (*p* < 0.05), ** Significant at 1% probability levels (*p* < 0.01), and *** Significant at 0.1% probability levels (*p* < 0.001).

**Table 4:** Results of the comparison between the mean effects of treatments with *P. gaudichaudianum* essential oil, *β*-caryophyllene, and the respective inclusion complexes and their concentrations on germination and root and aerial growth in *B. pilosa* and *L. multiflorum*.

Concentrations (µg mL^−1^)	Treatments	Germination (G)		Root Growth (RG)		Aerial Growth (AG)	
		*B. pilosa*	*L. multiflorum*	*B. pilosa*	*L. multiflorum*	*B. pilosa*	*L. multiflorum*
3000	GAU EO	3.2 ± 3.3 Bc	23.2 ± 15.8 Ab	2.3 ± 2.6 Bc	9.3 ± 5.7 Ab	0.0 ± 0.0 Ad	5.1 ± 3.0 Ac
	GAU IC	52.8 ± 18.8 Ab	19.2 ± 4.4 Bb	15.3 ± 2.4 Ab	13.2 ± 5.8 Aab	14.3 ± 0.7 Ac	13.0 ± 7.0 Ab
	*β*-CAR	86.4 ± 10.4 Aa	44.8 ± 15.6 Ba	20.3 ± 3.8 Ab	19.8 ± 6.2 Aa	22.3 ± 5.1 Ab	21.9 ± 6.8 Aa
	*β*-CAR IC	82.4 ± 6.1 Aa	28.8 ± 11.8 Bab	40.3 ± 1.8 Aa	13.2 ± 3.9 Bab	31.1 ± 5.3 Aa	13.6 ± 4.1 Bb
1500	GAU EO	4.0 ± 6.9 Bb	30.4 ± 14.3 Ab	1.8 ± 2.5 Bc	8.1 ± 1.6 Ab	0.0 ± 0.0 Ac	5.3 ± 1.7 Ac
	GAU IC	79.2 ± 7.7 Aa	35.2 ± 7.7 Bab	19.2 ± 1.1 Ab	17.1 ± 6.3 Aa	16.2 ± 4.3 Bb	22.1 ± 4.5 Aab
	*β*-CAR	92.0 ± 5.7 Aa	49.6 ± 8.8 Ba	24.1 ± 3.6 Ab	16.6 ± 5.4 Ba	26.4 ± 5.7 Aa	16.6 ± 7.3 Bb
	*β*-CAR IC	80.0 ± 9.4 Aa	44.8 ± 15.6 Bab	41.6 ± 4.2 Aa	15.9 ± 3.0 Ba	28.3 ± 7.9 Aa	27.4 ± 4.0 Aa
750	GAU EO	44.0 ± 25.3 Ab	29.6 ± 4.6 Ab	6.1 ± 3.8 Ac	9.8 ± 3.0 Ab	4.4 ± 4.1 Ac	5.5 ± 1.1 Ac
	GAU IC	79.2 ± 12.5 Aa	52 ± 11.0 Ba	24.4 ± 2.4 Ab	18.5 ± 5.9 Ba	19.2 ± 1.2 Ab	12.6 ± 3.3 Bb
	*β*-CAR	92.8 ± 8.2 Aa	48.8 ± 12.1 Ba	27.9 ± 3.4 Ab	20.7 ± 3.3 Ba	29.4 ± 4.4 Aa	24.8 ± 3.2 Aa
	*β*-CAR IC	88.0 ± 4.9 Aa	53.6 ± 14.6 Ba	35.4 ± 3.3 Aa	17.5 ± 5.9 Ba	31.4 ± 4.6 Aa	27.7 ± 3.3 Aa
375	GAU EO	76.0 ± 7.5 Aa	45.6 ± 6.7 Ba	12.2 ± 3.8 Ac	12.4 ± 6.2 Ab	8.4 ± 2.9 Ab	9.9 ± 2.3 Ab
	GAU IC	81.6 ± 12.2 Aa	50.4 ± 14.6 Ba	27.0 ± 0.7 Ab	17.7 ± 6.2 Bab	26.5 ± 3.3 Aa	16.4 ± 7.0 Bb
	*β*-CAR	83.2 ± 19.1 Aa	0.0 ± 0.0 Bb	13.0 ± 3.2 Ac	0.0 ± 0.0 Bc	0.0 ± 0.0 Ab	0.0 ± 0.0 Ac
	*β*-CAR IC	87.2 ± 8.7 Aa	62.4 ± 9.2 Ba	35.4 ± 3.3 Aa	24.1 ± 5.5 Ba	29.4 ± 6.4 Ba	35.5 ± 3.4 Aa
187.5	GAU EO	77.6 ± 8.8 Aa	42.4 ± 18.5 Ba	16.4 ± 3.8 Ac	14.9 ± 6.6 Abc	14.9 ± 3.4 Ab	14.2 ± 4.6 Ab
	GAU IC	84.0 ± 11.7 Aa	52.8 ± 11.1 Ba	27.0 ± 2.0 Ab	8.6 ± 1.8 Bc	25.9 ± 5.0 Aa	16.9 ± 4.5 Bb
	*β*-CAR	84.8 ± 10.4 Aa	53.6 ± 6.7 Ba	26.6 ± 5.9 Ab	18.9 ± 5.8 Bab	27.7 ± 3.6 Aa	29.7 ± 6.4 Aa
	*β*-CAR IC	80.0 ± 13.3 Aa	60.0 ± 7.5 Ba	37.1 ± 4.9 Aa	22.3 ± 9.7 Ba	26.1 ± 2.8 Ba	36.6 ± 1.5 Aa

Means followed by different letters, uppercase in columns (plants) and lowercase in rows (treatments), differ using Tukey’s test (*p* < 0.05). Mean ± standard deviation (*n* = 5).

**Table 5:** Mitotic index, genotoxicity, and mutagenicity observed in meristematic cells of *Lactuca sativa* roots exposed to *Psidium gaudichaudianum* essential oil (GAU EO) and *β*-caryophyllene (*β*-CAR).

Treatment	Concentration (µg mL^−1^)	Mitotic Index (%)	Genotoxicity (%)	Mutagenicity (%)
NC	-	8.16 ± 1.08 a	2.14 ± 0.32 a	0.00 ± 0.00 a
PC	-	8.57 ± 0.62 b	2.88 ± 0.25 b	0.00 ± 0.00 b
GAU EO	187.5	8.70 ± 0.78 ab	2.46 ± 0.71 ab	0.00 ± 0.00 ab
	375	8.96 ± 1.09 ab	2.24 ± 0.39 ab	0.02 ± 0.04 ab
	750	9.22 ± 1.29 ab	2.36 ± 0.50 ab	0.00 ± 0.00 ab
	1500	8.92 ± 0.57 ab	2.38 ± 0.29 ab	0.04 ± 0.05 ab
	3000	7.88 ± 1.13 ab	2.30 ± 0.51 ab	0.02 ± 0.04 ab
*β*-CAR	187.5	8.64 ± 0.84 ab	2.16 ± 0.55 ab	0.00 ± 0.00 ab
	375	4.82 ± 1.30	2.16 ± 0.46 ab	0.04 ± 0.05 ab
	750	7.2 ± 0.37 ab	2.22 ± 0.58 ab	0.00 ± 0.00 ab
	1500	7.22 ± 1.18 ab	1.96 ± 0.42 a	0.00 ± 0.00 ab
	3000	7.54 ± 1.24 ab	1.98 ± 0.36 a	0.00 ± 0.00 ab

Means that share different letters between lines differ statistically from each other with Dunnett’s test (*p* < 0.05). NC = negative control (acetone and tween 80 in aqueous medium); PC = positive control (methyl methanesulfonate).

**Table 6:** Mitotic, genotoxic, and mutagenic index observed in meristematic cells of *Lactuca sativa* roots exposed to the inclusion complex of essential *Psidium gaudichaudianum* (GAU IC) and *β*-caryophyllene (*β*-CAR IC).

Treatment	Concentration (µg mL^−1^)	Mitotic Index (%)	Genotoxicity (%)	Mutagenicity (%)
NC	-	9.36 ± 0.62 a	1.96 ± 0.38 a	0.00 ± 0.00 a
PC	-	8.57 ± 0.62 b	2.88 ± 0.25 b	0.00 ± 0.00 b
GAU IC	187.5	8.78 ± 0.61 ab	2.02 ± 0.30 a	0.00 ± 0.00 ab
	375	9.10 ± 0.16 ab	1.88 ± 0.38 a	0.04 ± 0.05 ab
	750	7.90 ± 0.80 b	1.48 ± 0.51 a	0.00 ± 0.00 ab
	1500	7.86 ± 0.82 b	1.72 ± 0.04 a	0.00 ± 0.00 ab
	3000	7.52 ± 0.49 b	1.52 ± 0.47 a	0.04 ± 0.05 ab
*β*-CAR IC	187.5	7.80 ± 0.69 b	1.48 ± 0.38 a	0.00 ± 0.00 ab
	375	8.54 ± 0.49 ab	1.82 ± 0.29 a	0.02 ± 0.04 ab
	750	8.80 ± 1.19 ab	1.82 ± 0.21 a	0.00 ± 0.00 ab
	1500	8.58 ± 0.44 ab	2.54 ± 0.57 ab	0.00 ± 0.00 ab
	3000	8.28 ± 1.12 ab	2.76 ± 0.36 b	0.00 ± 0.00 ab

Means that share different letters between lines differ statistically from each other with Dunnett’s test (*p* < 0.05). NC = negative control (2-hydroxypropyl-*β*-cyclodextrin in aqueous medium); PC = positive control (methyl methanesulfonate).

## Data Availability

Data of this study are available from the corresponding author when requested.

## Sample Availability

Samples of the compounds HP*β*CD, GAU EO, *β*-CAR, GAU IC, and *β*-CAR IC are available from the authors.
